# Sodium channel Nav1.7 expression is upregulated in the dorsal root ganglia in a rat model of paclitaxel-induced peripheral neuropathy

**DOI:** 10.1186/s40064-016-3351-6

**Published:** 2016-10-06

**Authors:** Yun Xiao, Zhongyuan Xia, Yang Wu, Bo Zhao

**Affiliations:** 1 Department of Anesthesiology, Renmin Hospital, Hubei University of Medicine, No.39, Chaoyang Road, Maojian District, Shiyan City, 442000 Hubei Province People’s Republic of China; 2 Department of Anesthesiology, Renmin Hospital of Wuhan University, Wuhan, 430060 Hubei Province People’s Republic of China

**Keywords:** Paclitaxel, Sodium channel Nav1.7, Dorsal root ganglion, Peripheral neuropathy

## Abstract

Paclitaxel-induced peripheral neuropathy is not completely known. Since the sodium channel Nav1.7 has been implicated in pain perception, and is upregulated in pain disorders, we investigated the effect of paclitaxel on Nav1.7 expression in rat dorsal root ganglion (DRG) neurons. Thirty Sprague-Dawley rats were administered either 2 mg/kg paclitaxel or vehicle on days 0, 2, 4 and 6. To evaluate nociceptive responses, paw withdrawal threshold (PWT) was measured by von Frey anesthesiometer on days 7, 14 and 21 after first paclitaxel administration. Expression of Nav1.7 in DRG was measured by real-time RT-PCR and Western blot. PWT was also measured in rats that received dorsal root ganglionic injection of either Nav1.7 antibody, neutralized Nav1.7 antibody or no injection (sham surgery) (n = 5/group). Average PWT was lower in animals administered paclitaxel than those administered vehicle at days 7 (*P* < 0.05), 14 (*P* < 0.01), and 21 (*P* < 0.01). DRG Nav1.7 mRNA and protein levels were higher in animals administered paclitaxel than those administered vehicle on days 7, 14 and 21 (all *P* < 0.05). PWT decrease was significantly correlated with increased Nav1.7 protein levels on days 7 (r = −0.88, *P* = 0.04), 14 (r = −0.46, *P* = 0.03) and 21 (r = −0.27, *P* = 0.01) after first paclitaxel administration. In animals that received sham surgery, neutralized Nav1.7 antibody or Nav1.7 antibody, PWTs were significantly reduced 7 days after first paclitaxel administration (all *P* < 0.05), but PWTs of animals that received Nav1.7 antibody were higher than those that received neutralized Nav1.7 antibody (*P* < 0.05). These results indicate that increased DRG Nav1.7 expression may be partially responsible for paclitaxel-induced peripheral neuropathy.

## Background

The chemotherapeutic drug paclitaxel, often used in the treatment of solid tumors, is reported to cause dose-dependent peripheral sensory neuropathy (Mielke et al. 2006), pain perceived to originate in the hands and feet (Loprinzi et al. 2007). The pathophysiological mechanisms underlying paclitaxel-induced hyperalgesia are complicated and not well understood (Jaggi and Singh 2012).

Primary sensory neurons in the dorsal root ganglion (DRG) receive signals produced by peripheral nerve endings, and incorporate and transmit these signals to the spinal cord. Because DRG neurons play a significant role in transmission of neural signals including pain, numerous studies have focused on its involvement in pathological pain (Krames 2014). Paclitaxel is reported to increase excitability of DRG neurons and affects expression of some pain-related genes in DRG neurons, such as TRPV1, an ion channel involved in the transmission and modulation of pain (Li et al. 2015; Hara et al. 2013). These studies demonstrate that DRG plays an essential role in regulating paclitaxel-induced peripheral neuropathy.

Voltage-gated sodium channels are crucial for electrogenesis in excitable cells. Nine pore-forming α-subunits of voltage-gated sodium channels (Navs) have been identified in mammals, termed Nav1.1 to Nav1.9 (Catterall et al. 2005). Nav1.7, encoded by the gene SCN9A, plays a crucial role in pain signal transduction in humans. Nav1.7 is selectively expressed in DRG neurons and sympathetic ganglia, particularly abundantly expressed in small-diameter DRG neurons, and preferentially expressed in nociceptors and evoked action potential firing in Aβ-fibers and C-fibers (Djouhri et al. 2003). At nerve endings, Nav1.7 produces resurgent currents in DRG neurons (Faber et al. 2012) and plays an important role in amplification of weak stimuli (Cummins et al. 1998). Nav1.7 can enhance subthreshold stimuli and increases the probability of neurons reaching their action potential threshold, and thus, Nav1.7 is considered to be a threshold channel.

Genetic studies have recognized Nav1.7 dysfunction in three different human pain disorders. Inherited gain-of-function missense mutations in Nav1.7 are found in erythromelalgia (IEM) and paroxysmal extreme pain disorder (PEPD) (Drenth and Waxman 2007; Fertleman et al. 2006). In contrast, recessively inherited loss-of-function mutations in SCN9A result in channelopathy-associated insensitivity to pain (CIP) (Goldberg et al. 2007).

Nav1.7 was also recently implicated in pain perception in some animal models used to study pain. Nav1.7 expression was found to be elevated in the DRG neurons of a diabetic neuropathy rat model (Chattopadhyay et al. 2008) and in a rat model of chronic constrictive injury (CCI) (Liu et al. 2012). Thus, in this study, we investigated whether paclitaxel-induced peripheral neuropathy involved in the expression changes of Nav1.7 in the DRG neurons of a model rat.

## Results

### Effect of paclitaxel on mechanical allodynia in rats

**Fig. 1:**
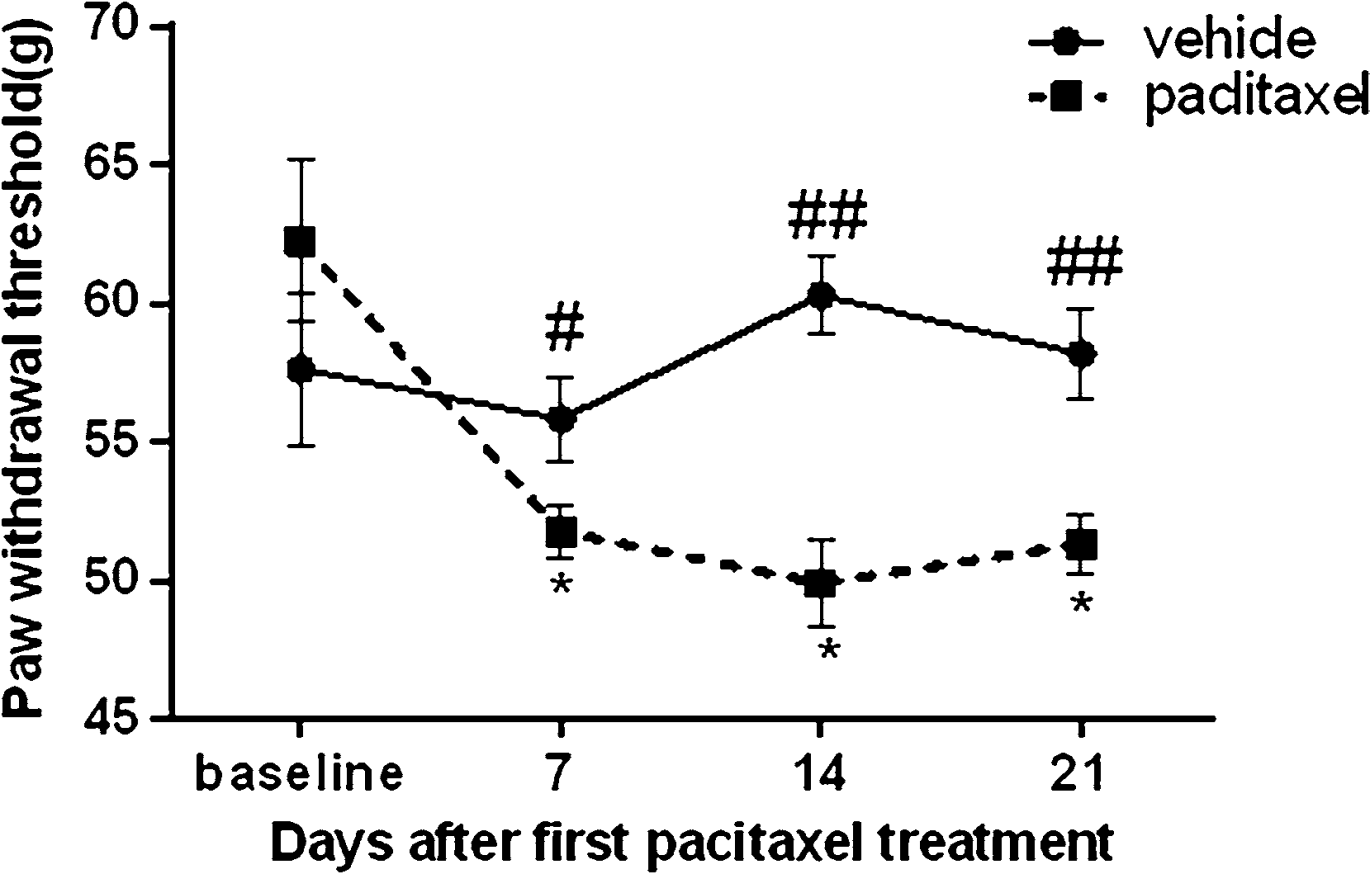
Effect of paclitaxel on mechanical allodynia in rats. Rats were administrated intraperitoneally at dose of 2 mg/kg paclitaxel or vehicle on days 0, 2, 4 and 6. The paw withdrawal threshold (PWT) to a static mechanical stimulus was assessed using a von Frey anesthesiometer. PWT was measured pre-injection (baseline) and on days 7, 14 and 21 after first paclitaxel administration. All data are expressed as mean ± SEM (n = 5/group/time point). *P < 0.05 versus baseline under the same treatment; ^#^ *P* < 0.05, ^##^ *P* < 0.01 versus paclitaxel group

We established a rat model of allodynia by administration of paclitaxel. The paw withdrawal threshold (PWT) did not differ significantly between the vehicle (57.61 ± 2.70 g) and paclitaxel (62.24 ± 2.90 g) groups at baseline (*P* = 0.901). In rats administered vehicle, the average PWT—to noxious mechanical stimulation did not differ 7, 14 and 21 days after first paclitaxel administration. In rats administered paclitaxel, the average PWT was lower than in animals administered vehicle at 7 (*P* < 0.05), 14 (*P* < 0.01), and 21 (*P* < 0.01) days after first paclitaxel administration, but the PWT measured on days 7, 14 and 21 did not differ significantly (all P > 0.05) (Fig. 1).

Normal weight gain was observed both in animals that received vehicle and those that received paclitaxel, and body weight did not differ between rats that received paclitaxel and vehicle (data not shown).

### Paclitaxel chemotherapy up-regulated Nav1.7 expression in DRG of rats

**Fig. 2:**
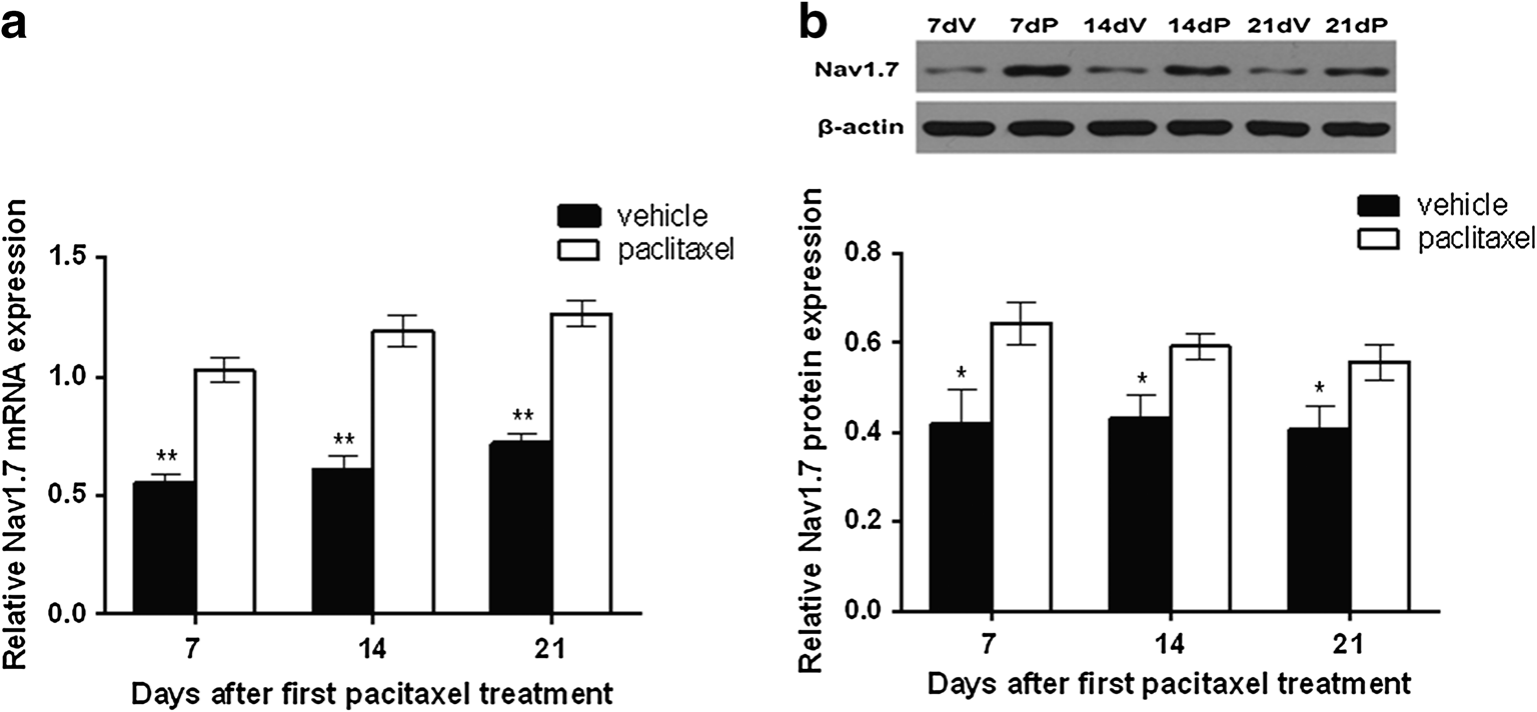
Up-regulation of dorsal ganglionic Nav1.7 expression after paclitaxel chemotherapy. **a** Nav1.7 mRNA expression in the dorsal ganglionic were determined by real-time RT-PCR on days 7, 14 and 21 after first paclitaxel administration. β-actin was used as the internal control. **b** Nav1.7 protein expression were measured by Western blot on days 7, 14 and 21 after first paclitaxel administration and expressed relative to the internal control, β-actin. All data are expressed as mean ± SEM (n = 5/group/time point). *P < 0.05 versus vehicle group

To investigate whether Nav1.7 in DRG was involved in paclitaxel-induced peripheral neuropathy, we measured expression of Nav1.7 mRNA and protein in DRG after paclitaxel administration. Expression of Nav1.7 mRNA in the DRG was higher in animals administered paclitaxel than those administered vehicle on days 7, 14 and 21 after first paclitaxel administration (all *P* < 0.01, Fig. 2a). The levels of Nav1.7 protein in the DRG were also higher in animals administered paclitaxel than those administered vehicle on days 7, 14 and 21 after first paclitaxel administration (all *P* < 0.05, Fig. 2b). As expected, no significant changes in Nav1.7 expression were observed in animals administered the vehicle, and levels of Nav1.7 mRNA and protein did not differ significantly between day 7 and 21 in animals administered paclitaxel.

### Association between pain sensitivity and Nav1.7 protein expression

To explore the association between pain sensitization and Nav1.7 protein expression within the paclitaxel-treated subjects, we assessed the correlations between PWT and Nav1.7 protein expression. We observed significant correlations between PWT decrease and increased Nav1.7 protein expression level on days 7 (r = −0.88, *P* = 0.04), 14 (r = −0.46, *P* = 0.03) and 21 (r = −0.27, *P* = 0.01) after first paclitaxel administration.

### Blocking function of dorsal root Nav1.7 partially attenuated paclitaxel-induced hyperalgesia in rats

**Fig. 3:**
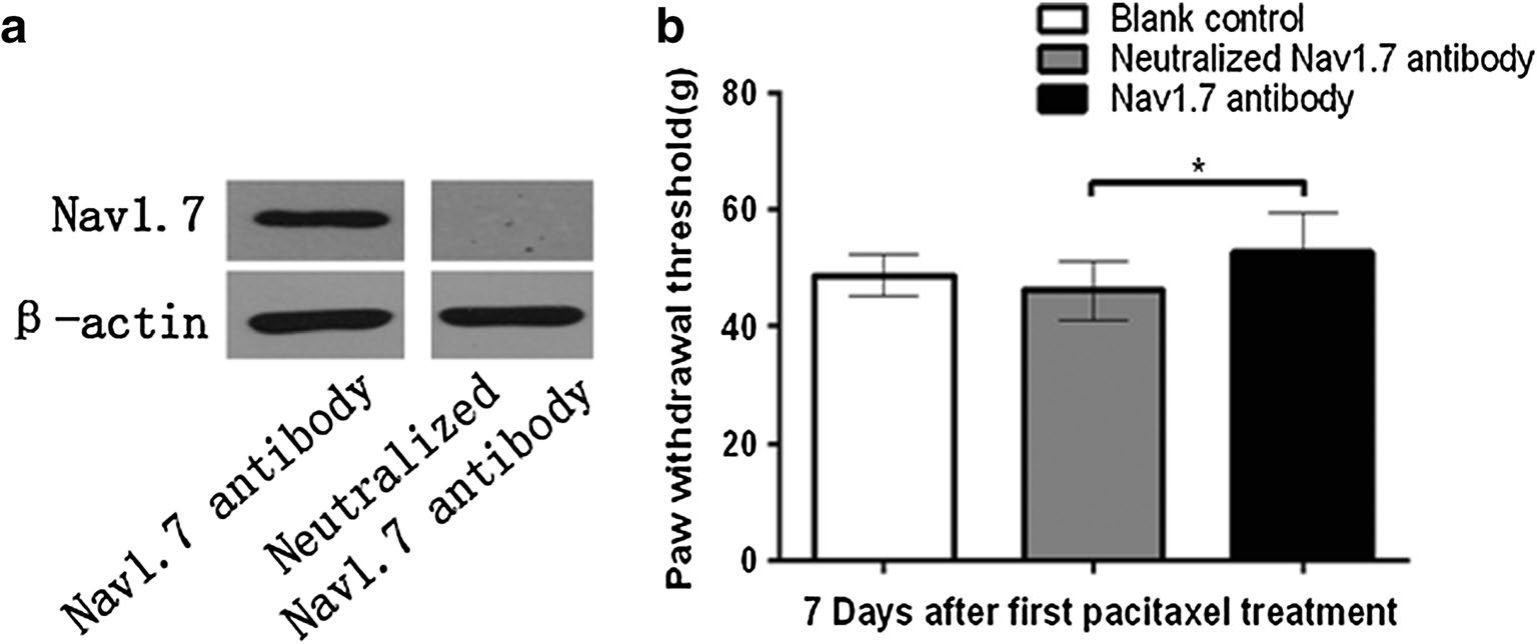
Blocking function of dorsal root ganglionic Nav1.7 partially attenuated paclitaxel-induced hyperalgesia in rats. Rats (n = 15) were equally divided into three groups, Sham surgery, neutralized antibody, and active antibody group, and received dorsal root ganglionic injection with 7 μL (5 μg) Nav1.7 antibody, neutralized Nav1.7 antibody, and underwent surgical exposure but no injection (sham surgery), respectively. The paclitaxel was intraperitoneal injected at dose of 2 mg/kg after 2 h of the DRGs microinjection on days 0, 2, 4 and 6. The PWT was measured at 1 day before-injection (baseline) and 7 days after first paclitaxel administration. **a** The neutralization efficiency was assessed using Western blotting with protein extracted from the rat ganglion tissues. β-actin was used as an inner control. The dorsal ganglionic Nav1.7 band can be detected by the Nav1.7 antibody, but not the neutralized Nav1.7 antibody. **b** Sham is compared with neutralized antibody and results indicate that surgery does not show a significant difference. And comparing neutralized antibody with active antibody, results indicate that PWT is significantly higher in functional antibody than neutralized one. All data are expressed as mean ± SEM (n = 5 each group). *P < 0.05 versus baseline under the same treatment; ^#^ *P* < 0.05 *vs.* Sham surgery, ^&^ *P* < 0.05 *vs.* neutralized Nav1.7 antibody 7 days after first paclitaxel administration

To further confirm whether dorsal root ganglionic Nav1.7 is involved in paclitaxel-induced neuropathic pain, we examined mechanical allodynia after injection of Nav1.7 antibody into DRGs to block ganglionic Nav1.7 function in vivo. The efficiency of Nav1.7 antibody neutralization was confirmed by Western blotting, and neutralized Nav1.7 antibody did not recognize Nav1.7, thus, was used as a negative control (Fig. 3a). Furthermore, we observed no differences between the baseline PWTs in sham surgery, neutralized Nav1.7 antibody and Nav1.7 antibody groups. However, 7 days after first paclitaxel administration, the PWTs were significantly reduced in animals that received sham surgery, neutralized Nav1.7 antibody or Nav1.7 antibody (all *P* < 0.05). Furthermore, the PWTs of animals that received Nav1.7 antibody were higher than those in the neutralized Nav1.7 antibody group (*P* < 0.05; Fig. 3b).

## Discussion

The mechanism by which Paclitaxel causes chemotherapy-induced peripheral neuropathy is poorly understood, but the Nav1.7 sodium channel has been implicated in perception of pain and is found to be upregulated in pain disorders (Drenth and Waxman 2007; Fertleman et al. 2006; Goldberg et al. 2007). The aim of this study was to investigate whether Nav1.7 in rat DRG neurons was involved in the paclitaxel-induced neuropathic pain. We found that in rats administered paclitaxel, the average PWT to noxious mechanical stimulation was lower than in animals administered vehicle at 7, 14 and 21 days after first paclitaxel administration. The results of this study are consistent with previously published findings (Zhang and Dougherty 2014).

Previous genetic studies have indicated that Nav1.7 is a key player in the processing of human pain, and Nav1.7 has become a focus of research as a therapeutic target for the treatment of pain. It was previously reported that Nav1.7 expression was increased in animal models of inflammation (Black et al. 2004; Chattopadhyay et al. 2008), diabetes (Chattopadhyay et al. 2008, 2011) and CCI (Liu et al. 2012), and a monoclonal antibody that targets Nav1.7 was reported to reduce inflammatory and neuropathic pain in mice (Lee et al. 2014). Our results provide the first direct evidence that paclitaxel chemotherapy induces a significant increase in Nav1.7 expression levels in DRGs. Furthermore, we examined nociceptive behavior after injection of Nav1.7 antibody into DRGs to block ganglionic Nav1.7 function in vivo. In animals that received sham surgery, administration of neutralized Nav1.7 antibody or Nav1.7 antibody, PWTs were significantly reduced 7 days after first paclitaxel administration. Additionally, the PWTs of animals that received Nav1.7 antibody were higher than those that received neutralized Nav1.7 antibody. These findings suggest that Nav1.7 is involved in the process by which paclitaxel induces neuropathic pain. However, as another study found that in a Nav1.7 conditional knockout mouse, expression of Nav1.7 was not required for oxaliplatin-induced pain and cancer-induced bone pain (Minett et al. 2014). These findings highlight that these different conditions may be caused by a variety of molecular mechanisms, and the role of Nav1.7 may vary in different pain models.

As expression of Nav1.8 and Nav1.9 overlaps with expression of Nav1.7 in DRG neurons (Strickland et al. 2008), to confirm that Nav1.7 exclusively was altered by Paclitaxel administration, we further measured expression of Nav1.8 and Nav1.9 mRNA in the DRG at each time point after first paclitaxel administration, but found no significant differences in expression of these channels between the vehicle and paclitaxel treatment groups at any time point (data not shown).

Expression of voltage-gated sodium channels is regulated by a variety of mediators. Expression of Nav1.7 was previously reported to be affected by levels of TNF alpha, MIP1 and 3, Fractalkine and cell adhesion molecule sICAM in the DRG (Galloway and Chattopadhyay 2013). In addition, nerve growth factor (NGF) and glial cell-derived neurotrophic factor (GDNF) could up-regulate expression of sodium channels in the DRG (Fjell et al. 1999; He et al. 2010). Further study will be required to characterize the mechanisms causing up-regulation of dorsal ganglionic Nav1.7 after paclitaxel administration.

## Conclusion

Dorsal ganglionic Nav1.7 was upregulated in rats administered paclitaxel. Blocking function of dorsal root ganglionic Nav1.7 partially attenuated paclitaxel-induced hyperalgesia in rats. Our observations suggest that Nav1.7 is involved in the process by which paclitaxel induced neuropathic pain, and may help in further understanding the mechanisms underlying neuropathic pain and in developing a new strategy to deal with paclitaxel-induced peripheral neuropathy.

## Methods

### Animals

Forty-five male Sprague-Dawley rats (aged between 8 and 12 weeks; body weight: 229.1 ± 22.1 g) were purchased from the experimental Animal Center of Hubei University of Medicine, China. The rats were housed under controlled conditions: temperature (22 ± 1 °C), with a 12-h light/dark cycle and free access to food and water. The experimental protocols were approved by the Animal Use and Care Committee of Hubei University of Medicine and were consistent with the Ethical Guidelines of the International Association for pain research.

### Drug administration

Paclitaxel (Bristol-Myers Squibb, Paris, France) was dissolved in cremophor EL: ethanol (1:1, Sigma), and diluted further with 0.9 % saline. 1 ml of a 6 mg/ml solution was administrated intraperitoneally (i.p.) at dose of 2 mg/kg on days 0, 2, 4 and 6. This dose was previously reported to induce mechanical allodynia/hyperalgesia in rats (Kawakami et al. 2012). Control rats received an equivalent volume of vehicle (cremophor EL:ethanol, 1:1) diluted with 0.9 % saline.

### Evaluation of mechanical allodynia

Mechanical allodynia was measured by recording the maximum pressure required to trigger hind paw withdrawal on day 0 (before paclitaxel administration, as baseline) and on days 7, 14 and 21 after first paclitaxel administration. The rats were allowed to habituate the testing chambers (22.0 × 15.0 × 12.5 cm) for 20 min. Mechanical allodynia was assessed using electronic von Frey anesthesiometer (0–90 g, electronic von Frey anesthesiometer, IITC Inc., Life Science Instruments, Woodland Hills, CA, USA) using a rigid tip, following a protocol adapted from that described by Vivancos et al. (2004). The pressure-meter consisted of a hand-held force transducer fitted with a 0.8 mm diameter polypropylene tip. The investigator applied the rigid tip perpendicularly to the mid-plantar surface of the hind paw with a gradual increase in pressure. A tilted mirror below the grid provided a clear view of the animal’s hind paw. The tests consisted of poking the hind paw to provoke a flexion reflex, followed by a clear flinch response after paw withdrawal. With the electronic pressure-meter, the maximum pressure of the stimulus was automatically recorded when the paw was withdrawn, as the pain threshold. The stimulation of the paw was repeated until the animal responded similarly (within 10 g) three times.

### Measurement of Nav1.7 expression in DRG

Five rats in each group were sacrificed by deep anesthesia with pentobarbital (50 mg/kg, i.p.) on days 7, 14 and 21 after first administration of paclitaxel or vehicle. DRGs (L_4–6_) were collected and processed for real-time RT-PCR and Western blotting, as described below.

### Quantitative real-time RT-PCR

RNA was extracted using TRIzol (Invitrogen, Carlsbad, CA). Reverse transcription and real-time PCR were performed as previously described (Wu et al. 2010). The rat β-actin primer sequences were as follows: sense: 5′-CGTTGACATCCGTAAAGACCTC-3′; anti-sense: 5′-TAGGAGCCAGGGCAGTAATCT-3′. The Nav1.7 primer sequences of were as follows: sense 5′-CGATGGGTCACGATTTCCTAC-3′; anti-sense 5′-CGTGAAGAATGAGCCGAAGAT-3′. In all cases, amplification was confirmed by the presence of a single peak in the melting temperature analysis and linear amplification throughout the PCR cycles. 2^−ΔΔCt^ was calculated to represent the relative mRNA expression of target genes. β-actin was used as an internal control.

### Western blot analysis

Protein was extracted from the DRG samples using a homogenizer in an ice-cold denaturing lysis buffer (25 mmol/L Tris–HCl, PH 7.5, 150 mmol/L Nacl, 5 mmol/L ethylenedianinetetraacetic acid, 1 % Triton X-100, 1 mmol/L PMSF, 1 μg/mL aprotinin, 1 μg/mL leupeptin), then the homogenate was centrifuged at 12,000×*g* for 20 min at 4 °C. The supernatant was collected, and protein concentration of the supernatant was determined via BCA Protein Assay Kit (Pierce, Rockford, USA). We loaded 40 μg of protein on each lane of 5 % SDS–PAGE gel, and transferred separated proteins to the polyvinylidene fluoride membrane (Millipore, Billerica, USA).

The membrane was blocked with 5 % non-fat dry milk in TBS-T (50 mmol/L Tris–HCl, pH 7.5, 140 mmol/L NaCl, 0.1 % Tween 20) overnight at room temperature (RT). The membranes were incubated with rabbit anti rat Nav1.7 primary antibody (1:500, Cell signaling Technology, MA, USA, #14573) for 3 h at RT, then incubated with HRP-anti-rabbit secondary antibody (1:4000, KPL,074-1506) for 1 h at RT. Blots were developed in ECL solution (Pierce, Rockford, USA) for 3 min, and exposed onto Kodak X-OMAT BT Film (Eastman Kodak, Rochester, USA) for 2 min. Densitometric analysis was performed using AlphaEaseFC software (Alpha Innotech, San Leandro, CA). Expression of Nav1.7 was normalized to the level of β-actin (1:10,000, TDY, Beijing, TDY051) in each sample.

### Dorsal root ganglionic injection of Nav1.7 antibody

The Nav1.7 antibody (57 μL of 0.7 μg/μL,Millipore, AB5390) was neutralized by incubation with 40 μg lyophilized antigen (Purified rat PN1 peptide, amino acids 446–460, Accession AAB50403, Millipore) for 1 h at RT according to the manufacturer’s instructions. The neutralization efficiency was assessed by Western blot, with protein extracted from the rat ganglion tissues.

We performed microinjection of Nav1.7 antibody into the DRGs using a previously described direct injection method (Fischer et al. 2011). Briefly, animals were anesthetized with pentobarbital (50 mg/kg, i.p.). To expose the DRG, an incision of approximately 3 cm was made in the skin just to the right of the dorsal midline, starting from the superior iliac crest. The fascia and muscles were separated, exposing the lateral aspect of the fourth and fifth lumbar (L4 and 5). The L4 and L5 spinal nerves, and the intervertebral foramina, from which they emerge, were exposed. Accessory processes that descend from the base of the transverse process were removed using a rongeur. The distal fourth and fifth DRGs were then exposed. The injections were performed using a pulled glass capillary injection tip with a diameter of 40–60 μm. Five rats received injection to the right side L4 and L5 DRGs, as previously described by Bi et al. (2013), with 7 μL (5 μg) Nav1.7 antibody (Millipore, AB5390). Five rats underwent the same treatment with neutralized Nav1.7 antibody, and five rats underwent surgical exposure but no injection (sham surgery). After the injection was completed, the wound was closed in layers.

The paclitaxel was intraperitoneally injected at dose of 2 mg/kg, 2 h after DRG microinjection on days 0, 2, 4 and 6. The PWT was measured at 1 day before-injection (baseline) and 7 days after first paclitaxel administration.

### Statistical analysis

Statistical analysis was performed with SPSS 17.0 (SPSS Inc., Chicago, IL, USA). All data are presented as mean ± SEM. All data of rat behavioral experiments were performed by two-way repeated-measure ANOVA with least significant difference (LSD) test for post hoc analysis. Other data were analyzed using independent sample *t* test, paired sample *t* test or one-way ANOVA with LSD test for post hoc analysis, as appropriate. Correlations between Nav1.7 protein levels with individual animal/time changes in von Frey PWT responses after paclitaxel administration were analyzed using the Pearson correlation test. A value of *P* < 0.05 was considered statistically significant.

## Abbreviations

DRG: dorsal root ganglion
IEM: erythromelalgia
PEPD: paroxysmal extreme pain disorder
NGF: nerve growth factor
GDNF: glial cell-derived neurotrophic factor
